# Influence of Conventional Resistance Training Compared to Core Exercises on Road Cycling Power Output

**DOI:** 10.7759/cureus.59371

**Published:** 2024-04-30

**Authors:** Sebastian Sitko, Isaac López-Laval, Rafel Cirer-Sastre

**Affiliations:** 1 Physiatry and Nursing, University of Zaragoza, Zaragoza, ESP; 2 National Institute for Physical Education of Catalonia, Universitat de Lleida, Lleida, ESP

**Keywords:** strength, core training, endurance, bike, power output

## Abstract

Conventional strength training and core exercises are commonly prescribed to improve cycling performance. Although previous studies have explored the utility of strength training in various cycling populations, this intervention has never been compared to core exercises. Thirty-six trained road cyclists were divided into three groups of 12 participants that performed either no strength training, conventional strength training, or core exercises, in all cases together with their regular cycling training during a 12-week period. Peak power outputs (POs) across different durations (five seconds, 60 seconds, five minutes, and 20 minutes) were recorded before and after the intervention. The results of the present study showed higher increases in relative PO with conventional strength training when compared to core training and no strength training for all measured durations: five-second Δ = 1.25 W/kg vs 0.47 W/kg and -0.17 W/kg; 60-second (Δ = 0.51 W/kg vs 0.13 W/kg and 0.02 W/kg; five-minute Δ = 0.22 W/kg vs 0.06 W/kg and 0.05 W/kg; and 20-minute Δ = 0.22 W/kg vs 0.07 W/kg and 0.06 W/kg. According to the data obtained in this study, conventional strength training is superior to core exercises, and no strength training was performed by trained road cyclists. Accordingly, it is recommended that this population incorporates strength training during their regular weekly workouts.

## Introduction

Road cycling is an endurance sport characterized by large training volumes and prolonged periods of moderate force production [1,2]. However, during decisive competition phases, short high-intensity efforts are necessary in order to perform [3]. This complex and variable requirement in force production may be addressed on the bike, but as previous evidence points out, the addition of conventional strength training may result in even better results. Strength training may delay the activation of less efficient type II fibers, improve neuromuscular efficiency, convert fast-twitch type IIX fibers into more fatigue-resistant type IIA fibers, and improve muscle-tendinous stiffness [4]. On the other hand, core training may have the potential to increase joint range and muscle extensibility, improve joint stability, enhance muscle performance, and optimize movement function [5].

The incorporation of strength training during the preparatory period of trained road cyclists has received increasing attention since the beginning of the century. Several authors have reported improvements in various markers of performance. After the addition of conventional strength training, increases in type IIA fiber proportions and decreases in type IIX; improvements in pedaling efficacy and cycling economy after fatigue; and increases in peak power output (PO), time trial performance, and PO at fixed blood lactate concentrations are some of the highlighted changes [6-8]. However, a previous study has also reported no effect of strength training when added to cycling training [9]. Although the consensus is towards a positive effect of these types of interventions, some degree of discrepancy still exists in the scientific and coaching field regarding the overall effect of strength training on cycling performance.

As road cycling is characterized by large training volumes performed under fixed postures in which the stabilizers and compensatory muscles of the trunk are being activated, the selective training of these muscle groups has received increased attention in the last years [2,10]. The practical coaching field has implemented training sessions with several exercises targeting the core muscles (transverse abdominis, multifidus, internal and external obliques, erector spinae, diaphragm, pelvic floor muscles, and the rectus abdominis [6]. Training of these muscles is seen as pivotal for efficient biomechanical function to maximize force generation and minimize joint loads in other endurance sports such as swimming, although the scientific evidence behind these interventions in cycling is lacking as of today [11,12].

Previous research that investigated the effect of strength training interventions on cycling performance evaluated laboratory parameters such as lactate profiles, oxygen uptake (VO2) kinetics, muscle fiber composition, and torque profiles [13]. Concretely, strength training interventions resulted in an earlier occurrence of peak torque values, higher POs at fixed blood lactates, transitions towards slower twitch muscle fiber types, and improvements in gross efficiency represented as the power-to-VO2 relationship [14,15]. Although these values are useful for the prediction of cycling performance, during the last years, attention has focused towards the power profile, which allows us to accurately predict performance after the analysis of POs registered with a mobile power meter [3]. This method allows an accurate, flexible, and inexpensive longitudinal tracking of performance without resorting to the laboratory. Previous research has shown that five-second, 60-second, five-minute, and 20-minute intervals can be used to track neuromuscular and glycolytic changes, maximal oxygen consumption, and maximal metabolic stable state, respectively [16-18].

Given the relative uncertainty regarding the true effect of conventional strength training on cycling performance, the lack of previous studies regarding the utility of core training, and the easy implementation of power profiling as a valid tool to monitor changes in performance experienced after strength training, the objective of the current study was to compare the power profile of trained road cyclists after 12 weeks of a) conventional strength training, b) core training, and c) no strength training during the preparatory phase of the annual training plan.

## Materials and methods

The current study was structured as an exploratory intervention aimed at assessing training effects. A test protocol, outlined below, was conducted both at baseline (pre-intervention) and after a 12-week intervention period (post-intervention). The study duration, spanning from November to January, encompassed the off-season and the initial phase of preparation leading up to the competitive season.

Participants

Thirty-six cyclists volunteered, with mean (standard deviation) (range) characteristics as follows: age = 28.8 (4.2) (21, 37) years; height = 179.3 (5.1) (169, 190) cm; body mass = 69.1 (4.5) (60, 80) kg. Random allocation divided participants into three groups of n = 12: cycling, cycling & core, and cycling & strength. All participants trained at the same facility underwent assessments using identical equipment and were evaluated by the same researcher. Inclusion criteria included possessing a cycling license (World Tour, Elite/U23, Masters, or recreational), the absence of surgical procedures or injuries in the preceding six months, and no use of performance-enhancing supplements or drugs during the same period. Following informed consent and completion of a health-screening questionnaire, participants were enrolled in the study, adhering to ethical guidelines outlined in the 2013 Declaration of Helsinki and approved by the Research Ethics Committee of the autonomous region of Aragon, Spain (PI23/131) [19]. Grouped participant characteristics are presented in Table 1.

**Table 1:** Summary of participant characteristics. Note: Data are expressed as mean (standard deviation) (range)

Variable	Cycling (n=12)	Cycling & Core (n=12)	Cycling & Strength (n=12)
Age (years)	30 (4) (22, 36)	29 (5) (21, 37)	28 (4) (22, 36)
Body height (cm)	180 (6) C170, 190C	179 (5) (169, 187)	179 (5) (170, 185)
Body mass (kg)	69 (6) (60, 80)	69 (3) (62, 73)	70 (4) (60, 74)

Procedures

Subjects underwent anthropometric evaluation and cycling tests before and after the 12-week intervention period, conducted within the morning hours (between 10:00 AM and 12:00 PM) to mitigate diurnal hormonal fluctuations. Data collection occurred under consistent environmental conditions (17-18°C, 45-55% relative humidity). Cycling tests were performed on personal bikes mounted on a Tacx Neo 2T Smart bike trainer (Tacx International, Rijksstraatweg, Netherlands), with PO measured using Favero Assioma pedals [16]. Maximal oxygen consumption was estimated using a formula based on relative PO obtained during a five-minute interval [20].

In order to obtain the power profile of each participant, subjects performed an adaptation of the testing protocol suggested by Allen et al. as follows: (a) 20 minutes at a self-selected easy intensity; (b) three one-minute fast pedaling accelerations (100-105 rpm) with a one-minute recovery between efforts; (c) five minutes at a self-selected easy intensity; (d) one all-out five-second sprint; (e) five minutes at a self-selected easy intensity; (f) one-minute all-out effort; (e) five minutes at a self-selected easy intensity; (g) five-minute all-out effort; (h) 10 minutes at a self-selected easy intensity and five minutes of resting [2]. The main part of the test consisted of a 20-minute maximal effort, where subjects were asked to produce the highest mean PO possible for this duration and adopt their personal pacing strategies [2,21-23]. Participants were familiarized with the protocol as it was commonly included in their normal testing routine. They could also view their progress on a computer monitor and were provided with information regarding time to completion and gear choice. All other information was blinded, no verbal encouragement was provided, and water was allowed ad libitum. functional threshold power (FTP) was determined as 95% of the mean PO of the 20-minute effort. Maximal POs for each selected duration were registered in relative values (W/kg) considering the weight obtained during the anthropometric evaluation.

Anthropometric evaluation

Body mass and fat mass were assessed using the electrical impedance method (BC-602; Tanita Co., Tokyo, Japan) in the morning, while height was measured using a SECA 214 stadiometer (Seca; Hamburg, Germany), graduated up to 1 mm.

Endurance training

All participants performed the same training sessions during the 12 weeks of the intervention period. Training was prescribed based on six power zones relative to the FTP, which was calculated by subtracting 5% from the maximal 20-minute PO obtained in the baseline testing [2]. The power zones were set up as follows: <55% Zone 1; 56-75% Zone 2; 76-90% Zone 3; 91-105% Zone 4; 106-120% Zone 5; and >121% Zone 6 [2]. Participants performed four weekly sessions riding in Zone 2 (two hours on Tuesday, two hours on Thursday, four hours on Saturday, and four hours on Sunday). Compliance was verified by analyzing files obtained from the bike computer of the participant. A total of 34 out of 36 participants (94%) achieved a compliance of >90% when the training stress score of the full intervention period was assessed.

Strength training

The strength training exercises were based on previous research and in the following order (half squat, leg press with one leg at a time, one-legged hip flexion, and ankle plantar flexion) and were performed twice weekly (Monday and Wednesday) [4]. Three-minute rests were allowed between sets. All cyclists were supervised by an investigator at all workouts during the first two weeks and thereafter at least once every second week throughout the intervention period. During the 12 weeks of the intervention period, cyclists trained with six repetitions maximum (RM) sets until failure. The cyclists were encouraged to increase their RM loads after four and eight weeks of the intervention period, and they were allowed assistance on the last repetition. The number of sets in each exercise was always three. Over the entire training period, one session was not performed due to illness.

Core training

The core exercises (glute bridge, abdominal plank, and prone back extension) were performed as recommended in a previous study designed with cyclists [24]. The glute bridge and prone back extension consisted of a two-second concentric phase, a two-second isometric phase, and a four-second eccentric phase. Both exercises incorporated 10 repetitions for each set. The abdominal plank was maintained for 30 seconds. Eight sets of each exercise were performed with a 60-second rest in between. These exercises were performed twice weekly (Monday and Wednesday). All cyclists were supervised by an investigator at all workouts during the first two weeks and, thereafter, at least once every second week throughout the intervention period. Over the entire training period, two sessions were not performed due to illness.

Statistical analyses

Data are described as mean (standard deviation) (range). Baseline differences between the three groups were assessed by univariate analyses of variance (ANOVA). To compare the effectiveness of each intervention, a one-way between-groups analysis of covariance (ANCOVA) was conducted. The base analysis model was built with group allocation as the independent variable and the absolute change (Δ = post-pre) as the dependent variable, with the corresponding baseline scores as covariates. Prior to the analysis, the assumptions of linearity, homogeneity of regression slopes, normality of residuals, homogeneity of variances, and absence of outlier values were inspected. Accordingly, the following decisions were made: five-minute RPO violated slope homogeneity and was heteroscedastic, and, consequently, its modeling included an interaction term with group-by-baseline data and the White-Huber heteroscedasticity correction. Additionally, 60-second RPO had baseline differences, and, consequently, its modeling included an interaction term with group-by-baseline data. Post-hoc pairwise comparisons were based on model-estimated marginal means and reported as estimated means and/or mean changes (Δ) and their 95% confidence interval (CI). Effect sizes for ANCOVA terms were reported as partial eta 2 (h2P), and for pairwise comparisons as Cohen’s d, in both cases with their respective 95% CI. Analyses were performed using R (version 4.2.2; R Development Core Team, Vienna, Austria), and statistical significance was assumed when p < 0.05.

## Results

Anthropometric data

At baseline, all groups had similar body mass (F(2, 33) = 0.06, p = 0.94), fat mass (F(2, 33) = 3.07, p = 0.06), body mass index (F(2, 33) = 1.21, p = 0.31), and VO2 max (F(2, 33) = 1.11, p = 0.34). Changes after training were also comparable among groups in body mass (F(2, 32) = 0.12, p = 0.89), fat mass (F(2, 30) = 0.94, p = 0.402), and body mass index (F(2, 32) = 0.026, p = 0.97). By contrast, VO2 max improvements were different among groups (F(2, 32) = 6.84, p = 0.003, h2P = 0.30, 95% CI (0.08, 1.00)), being higher in the cycling & strength group (Δ = 2.14 mL/min/kg, Δ 95% CI (1.4, 2.47) mL/min/kg) compared with the cycling-only group (Δ = 0.35 mL/min/kg, Δ 95% CI (-0.27, 1.21) mL/min/kg, t(32) = 3.22, p = 0.008, d = 1.14, d 95% CI (0.38, 1.88)) and the cycling & core group (Δ = 0.36 mL/min/kg, Δ 95% CI (-0.2, 1.25) mL/min/kg, t(32) = 3.2, p = 0.008, d = 1.13, Δ 95% CI (0.38, 1.87)).

Main relative PO (RPO) differences

**Figure 1:**
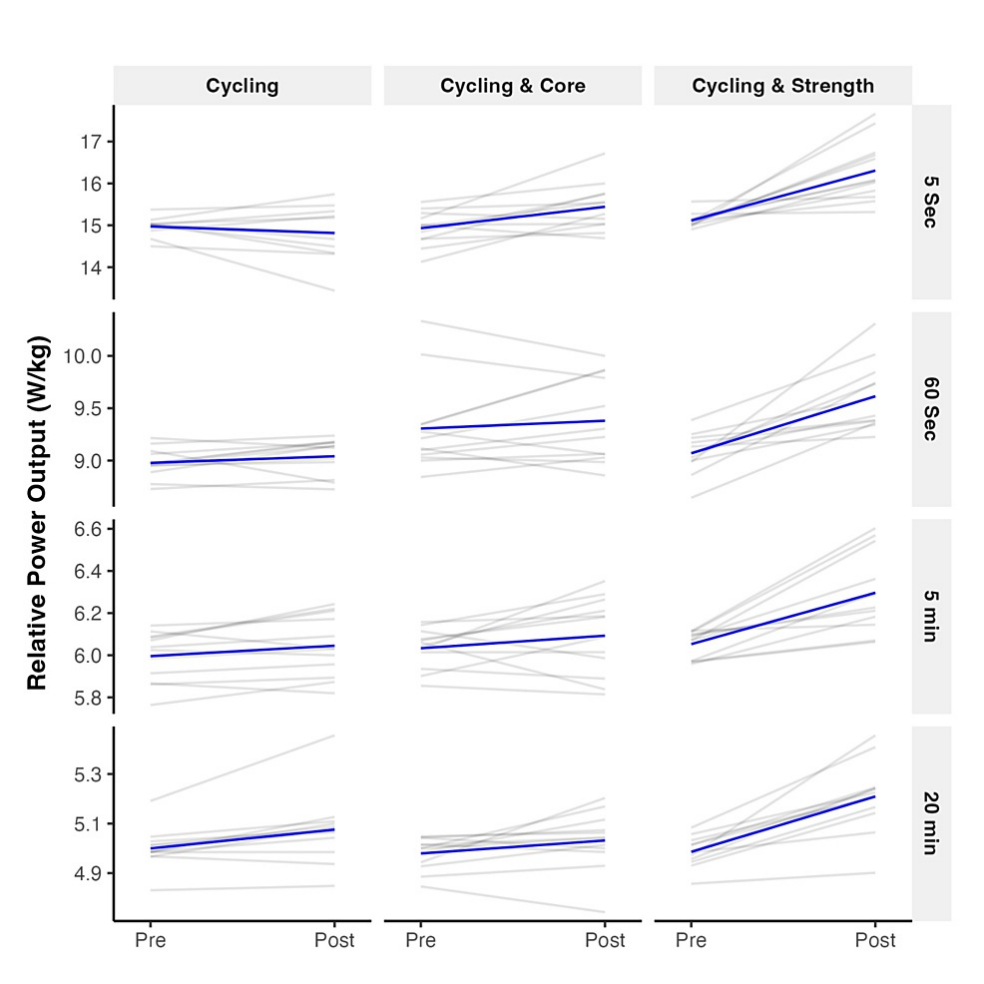
Individual changes in relative power output by test.

Individual RPO is summarized in Table 2, and individual trends can be inspected in Figure 1. There were no group differences in baseline five-second RPO (F(2, 33) = 1.39, p = 0.263), five-minute RPO (F(2, 33) = 1.11, p = 0.34), and 20-minute RPO (F(2, 33) = 0.27, p = 0.763), but a statistically significant and large main effect of group in 60-second RPO (F(2, 33) = 4.16, p = 0.024, h2P = 0.20, 95% CI (0.02, 1.00)). Adjusted by baseline data, RPO improvements after the intervention were different among groups in all tests: five-second RPO (F(2, 32) = 14.09, p < 0.001, h2P = 0.47, 95% CI (0.24, 1.00)), 60-second RPO (F(2, 30) = 11.96, p < 0.001, h2P = 0.44, 95% CI (0.20, 1.00)), five-minute RPO (F(2, 32) = 5.77, p = 0.008, h2P = 0.28, 95% CI (0.06, 1.00), and 20-minute RPO (F(2, 32) = 11.72, p < 0.001, h2P = 0.42, 95% CI (0.19, 1.00)).

**Table 2:** Participants' performance in the relative power output tests (RPO) by group and time. Note: Data are expressed as mean (standard deviation).

	Cycling Only		Cycling & Core		Cycling & Strength			
Variable	Pre	Post	Pre	Post	Pre	Post	Adj. Group dif.	Adj. Time dif.
5-sec RPO (W/kg)	14.97 (0.22)	14.82 (0.63)	14.93 (0.41)	15.44 (0.57)	15.12 (0.17)	16.31 (0.73)	F = 1.39, p = 0.263	F = 14.09, p < 0.001
60-sec RPO (W/kg)	8.98 (0.14)	9.04 (0.17)	9.31 (0.44)	9.38 (0.41)	9.07 (0.19)	9.61 (0.32)	F = 4.16, p = 0.024	F = 11.96, p < 0.001
5-min RPO (W/kg)	6 (0.12)	6.04 (0.14)	6.03 (0.09)	6.09 (0.18)	6.05 (0.06)	6.3 (0.19)	F = 1.11, p = 0.34	F = 5.77, p = 0.008
20-min RPO (W/kg)	5 (0.08)	5.08 (0.14)	4.98 (0.06)	5.03 (0.12)	4.99 (0.06)	5.21 (0.14)	F = 0.27, p = 0.763	F = 11.72, p < 0.001

Post-hoc group contrasts by variable

**Figure 2:**
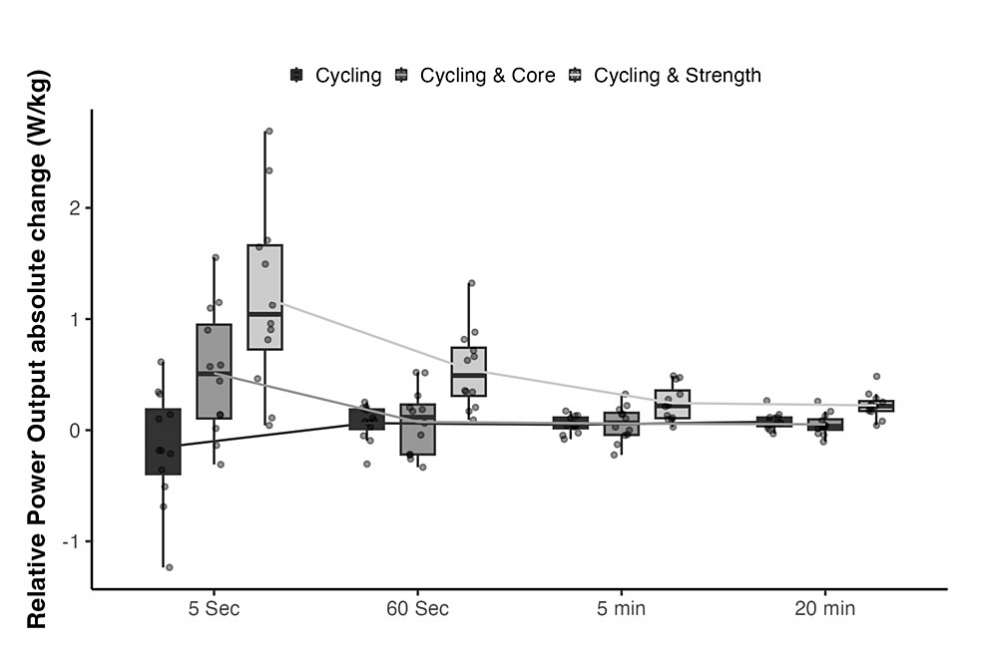
Absolute mean changes in relative power output by group and test.

Absolute mean changes are provided in Figure 2. Statistical contrasts revealed that, adjusted by baseline, the mean five-second RPO improvements were higher in the cycling & strength group (Δ = 1.25 W/kg, Δ 95% CI (0.86, 1.64) W/kg) compared to the cycling-only (Δ = -0.17 W/kg, Δ 95% CI (-0.55, 0.21) W/kg, t(32) = 5.3, p < 0.001, d = 1.88, d 95% CI (1.04, 2.7)) and the cycling & core group (Δ = 0.47 W/kg, Δ 95% CI (0.09, 0.85) W/kg, t(32) = 2.86, p = 0.02, d = 1.01, d 95% CI (0.27, 1.74)). Additionally, five-minute RPO was higher in the cycling & core group compared to the cycling-only group (t(32) = 2.46, p = 0.049, d = 0.87, d 95% CI (1.14, 1.59)).

Improvements in 60-second RPO were also higher in the cycling & strength group (Δ = 0.51 W/kg, Δ 95% CI (0.34, 0.67) W/kg) compared to the cycling-only group (Δ = 0.02 W/kg, Δ 95% CI (-0.21, 0.24) W/kg, t(30) = 3.58, p = 0.003, d = 1.31, d 95% CI (0.51, 2.09)) and cycling & core group (Δ = 0.13 W/kg, Δ 95% CI (-0.04, 0.3) W/kg, t(30) = 3.22, p = 0.008, d = 1.18, d 95% CI (0.39, 1.94)).

RPO in five-minute also improved more in the cycling & strength group (Δ = 0.22 W/kg, Δ 95% CI (0.14, 0.32) W/kg) compared to the cycling-only group (Δ = 0.05 W/kg, Δ 95% CI (-0.04, 0.14) W/kg, t(30) = 2.9, p = 0.018, d = 1.06, d 95% CI (0.29, 1.82)) and the cycling & core group (Δ = 0.06 W/kg, Δ 95% CI (-0.02, 0.14) W/kg, t(30) = 2.8, p = 0.023, d = 1.02, d 95% CI (0.26, 1.78)).

Finally, the mean 20-minute RPO improvement was greater in the cycling & strength group (Δ = 0.22 W/kg, Δ 95% CI (0.17, 0.28) W/kg) compared to the cycling-only group (Δ = 0.07 W/kg, Δ 95% CI (0.02, 0.13) W/kg, t(32) = 3.98, p = 0.001, d = 1.41, d 95% CI (0.62, 2.17)) and the cycling & core (Δ = 0.06 W/kg, Δ 95% CI (0.00, 0.11) W/kg, t(32) = 4.37, p < 0.001, d = 1.54, d 95% CI (0.75, 2.32)).

## Discussion

The objective of the current study was to compare the effects of conventional strength training, core training, and no strength training in trained road cyclists. The main findings were as follows: a) conventional strength training was superior to core training and no training for the improvement of five-second, 60-second, five-minute, and 20-minute POs; b) core training was superior to no training for the improvement of five-second POs only; and c) there were no differences in body composition changes across the three groups, although the VO2 max increase was larger in the conventional strength training group.

To the best of the author's knowledge, this is the first study to compare conventional strength versus core training for the improvement of road cycling performance. The results support previous evidence that suggested the general utility of conventional strength exercises when contextualized into a concurrent strength and endurance training program [4,14,15]. This was not the case for core exercises, a lack of effect suggesting that these types of interventions do not benefit road cyclists. Several theoretical benefits have been suggested for core stability and strength training: increases in joint range and muscle extensibility, improvements in joint stability, enhanced muscle performance, and optimized movement function are among the most reported [6]. Some of these possible benefits could be of interest to road cyclists given that most of them translate into a better exercise economy [1,25]. Given that an improvement in exercise economy may not necessarily translate into an increase in mean maximal POs, the current study could have overlooked this hypothetical benefit, which should be explored in the future [7]. Despite this possibility, most cyclists have limited time to train and need to limit cycling time in order to incorporate some kind of strength training into their routine. Accordingly, the results of the current study suggest most cyclists would make better use of their time by performing conventional strength exercises rather than core training.

Core training resulted in an increase in five-second RPO when compared to no strength training. This finding should be further contextualized as the no-strength training group actually decreased its sprinting power after the 12-week intervention period. This could be related to the fact that no sprints were performed during the study. Sprinting power is not normally the main objective during the preparatory period of the annual training cycle. Given the time and effort required to perform the core training sessions, the cost/benefit ratio does not seem very attractive in this case and the time of the season [10,18]. Theoretically, an improvement in core strength and stability could result in an optimized movement pattern during sprinting, which would result in an increase in PO during this race moment [10]. However, as of today, it is impossible to know whether an improvement in core strength and stability is the cause of this effect: there is a lack of a gold standard method for measuring core stability and strength during sporting movements. Further, few studies have observed any performance enhancement in sporting activities despite observing improvements in core stability and core strength following a core training program [6]. Finally, there are no official guidelines, nor scientific evidence regarding the best intensity, volume, and distribution of core training sessions for the improvement of sporting performance. Accordingly, the intervention used in the current study, although based on previous research with cyclists, may have produced an insufficient stimulus to produce any measurable change in performance [26]. Given all the above, the results of the current study do not support core training as a time-efficient strategy to improve road cycling performance.

No significant differences in the anthropometric characteristics were observed after 12 weeks of intervention. This finding is interesting given that previous research has reported increases in muscle mass, changes in fiber type, and alterations of body mass after conventional strength training performed with heavy weights [1,4,17]. As the main interest of the present study was to assess the RPO after the intervention period, changes in body mass and fat mass, but not muscle mass, were tracked. Therefore, the evolution of the specific components of body composition cannot be discussed in this case. Given the lack of difference in the evolution of body mass and the clear increase in the VO2 max in the conventional strength training group, this change can be attributed to the increase in absolute PO observed in this group [25]. Improvements in either fractional or maximal oxygen utilization have been reported in previous research and are probably related to postponed activation of less efficient type II muscle fibers, conversion of type IIX fibers into more fatigue-resistant IIa fibers, and increased muscle mass and rate of force development [17,26]. Given these results, conventional strength training is an interesting addition to cycling training in order to improve the VO2 max.

The current study presents several limitations that should be addressed in future research. First, for obvious reasons, participants could not be blinded and accordingly were exposed to the placebo effect. Concretely, past research shows that participants’ belief in the efficacy of an intervention may influence findings in sports science research [27]. The conventional strength training group could have been exposed to this effect and, consequently, could have reported better results [28]. Future research utilizing a single-blind design may potentially reduce this limitation. Second, the sample was composed of participants of different cycling levels. The sample size was insufficient to compare across performance groups and try to extrapolate whether the findings obtained in the current study could be applied to all cyclist irrespective of their level. Finally, several important physiological parameters such as lactate or heart rate were not monitored in this study. This is important as improvements in relative blood lactate values or cardiac drift can be obtained even in the absence of increases in PO [29].

## Conclusions

Road cyclists are often limited by their time availability in order to choose the best training plan for maximal performance optimization. The incorporation of strength training sessions into the weekly training routine may reduce the time spent on the bicycle, and, accordingly, this decision should not be taken lightly. The present study suggests that 12 weeks of conventional strength training added to cycling during the preparatory phase of the season results in increased POs over the entire power curve and improves the VO2 max. These results were superior to those obtained from core training, which only increased PO during sprints. Given the notable performance gains and great adherence observed in the current study, it is recommended that cyclists include bi-weekly strength training sessions in their preseason. The exercises should target the main muscles activated during the pedal stroke (half squat, leg press with one leg at a time, one-legged hip flexion, and ankle plantar flexion) with intensities of six RM and three sets per exercise.
